# Greater Adolescent Cognitive Ability Linked to Lower Risk of Earlier Mortality

**DOI:** 10.1093/geroni/igab046.1996

**Published:** 2021-12-17

**Authors:** Tara Gruenewald, Molli Grossman, Catalina Zavala, Thalida Arpawong, Gwen Fisher, Kenneth Shultz, Margaret Gatz, Carol Prescott

**Affiliations:** 1 Chapman University, Orange, California, United States; 2 University of Southern California, Los Angeles, California, United States; 3 Colorado State University, Fort Collins, Colorado, United States; 4 California State University, San Bernardino, San Bernadino, California, United States

## Abstract

There have been few investigations of the role that adolescent cognitive ability might play in predicting physical resilience across the life course, including decreased risk of early mortality. Our limited knowledge of how multiple cognitive ability domains shape trajectories of longevity is due, in part, to a lack of aging cohorts with early life cognitive assessments, and family data that allow for examination of shared family and genetic characteristics that may play a role in cognitive ability-health links. We capitalized on data from the 1960 Project Talent high school cohort (n>360,000, born 1942-1946) and mortality data (n=22,584; 5,497 deceased) collected as part of two recent follow-ups, the Project Talent Twin & Sibling Study and the Project Talent Aging Study, to examine these potential associations. In 1960, ability was assessed in multiple cognitive domains (e.g., general aptitude, quantitative, reasoning). Mortality status was ascertained through 2016. Binary logistic generalized estimating equations with race, age, sex, and adolescent family SES covariates, indicated that each 1 standard deviation higher ability in multiple cognitive domains in adolescence predicted lower odds of earlier mortality (ORs of 0.79 - 0.87). Co-sibling control models indicated a similar pattern, suggesting that benefits associated with higher cognitive performance do not simply reflect shared environmental and genetic background, but may represent a direct protective effect. These findings indicate that better performance in multiple cognitive domains in adolescence, above and beyond the influence of genetic and family environmental factors, may be or point to modifiable protective factors against risk of early mortality.

